# Supplementary material for: Differences in meiofauna communities with sediment depth are greater than habitat effects on the New Zealand continental margin: implications for vulnerability to anthropogenic disturbance
Source: PeerJ. 2016 Jul 5;4:e2154. doi: 10.7717/peerj.2154 (PMC4941793; doi:10.7717/peerj.2154)
Supplement: Supplemental Information 1 — [STD = Standard deviation of depth based on 3, 5, 7, 15 grid cell focal mean, Slope STD = Standard deviation of slope, Vrm = terrain rugosity, range = depth range, curvature = change of the slope, profile curvature = curvature of the surface in the direction of the slope, plan curvature = curvature of the surface perpendicular to the slope direction]. [file peerj-04-2154-s001.docx]

Table S1. List of final environmental variables that were used in the DistLM analysis and the correlated variables that were removed prior to analysis of meiofauna for Hikurangi Margin and Bay of Plenty study region. [STD = Standard deviation of depth based on 3, 5, 7, 15 grid cell focal mean, Slope STD = Standard deviation of slope, Vrm = terrain rugosity, range = depth range, curvature = change of the slope, profile curvature = curvature of the surface in the direction of the slope, plan curvature = curvature of the surface perpendicular to the slope direction].

| **Hikurangi Margin** | | **Bay of Plenty** | |
| --- | --- | --- | --- |
| Final variables | Correlated variables removed | Final variables | Correlated variables removed |
| Latitude | Longitude | %OC | %N |
| Surface water chlorophyll concentration | Longitude | Phaeopigment | Sediment Chl a |
| %OC | %N | Sorting | Mean particle size |
| Phaeopigment | Sediment Chl a | %silt/clay | Particle size diversity |
| Mean particle size | Sorting | Vrm05 | Vrm03 |
| Particle size diversity | %silt/clay | STD07 | STD15 and STD05 |
| Vrm05 | Vrm03 | STD05 | STD03 |
| STD15 | STD07 | Slope STD07 | Slope STD15 |
| STD07 | STD05 | Slope STD05 | Slope STD07 and slope STD03 |
| STD05 | STD03 | Range 07 | Range 15 and Range 05 |
| Slope STD15 | Slope STD07 | Range 05 | Range 03 |
| Slope STD03 | Slope STD05 | Plan curvature | Curvature |
| Range 07 | Range 15 | Depth |  |
| Range 05 | Range 07 and  Range 03 | Fishing intensity | |
| Curvature | Plan curvature | Slope |  |
| Depth |  | Profile curvature | |
| %CaCO_3_ |  | Longitude |  |
| %OM |  | Latitude |  |
| Skewness |  | Surface chlorophyll concentration | |
| Kurtosis |  | %CaCO_3_ |  |
| Fishing intensity | | %OM |  |
| Slope |  | Skewness |  |
| Profile curvature | | Kurtosis |  |
