# Supplementary material for: Differences in meiofauna communities with sediment depth are greater than habitat effects on the New Zealand continental margin: implications for vulnerability to anthropogenic disturbance
Source: PeerJ. 2016 Jul 5;4:e2154. doi: 10.7717/peerj.2154 (PMC4941793; doi:10.7717/peerj.2154)
Supplement: Supplemental Information 2 — [Av.Value = average environmental variable value, Av.Sq.Dist = average dissimilarity, Sq.Dist/SD = Dissimilarity/Standard Deviation, Contrib% = % contribution to overall dissimilarity, Cum.% = % cumulative dissimilarity]. Higher average value are shown in bold. [file peerj-04-2154-s002.docx]

Table S2. SIMPER analysis results showing environmental variables accounting for regional dissimilarity between the Hikurangi Margin and Bay of Plenty study regions (cut-off applied at 70% contribution).[Av.Value = average environmental variable value, Av.Sq.Dist = average dissimilarity, Sq.Dist/SD = Dissimilarity/Standard Deviation, Contrib% = % contribution to overall dissimilarity, Cum.% = % cumulative dissimilarity]. Higher average value are shown in bold.

| Variable | Av.Value | Av.Value | Av.Sq.Dist | Sq.Dist/SD | Contrib% | Cum.% |
| --- | --- | --- | --- | --- | --- | --- |
|  | Hikurangi Margin | Bay of Plenty |  |  |  |  |
| Surface water chlorophyll concentration | **0.96** | -0.8 | 3.56 | 1.43 | 12.4 | 12.4 |
| Phaeopigment | **0.87** | -0.73 | 3.34 | 1.02 | 11.65 | 24.05 |
| %OM | **0.76** | -0.63 | 2.94 | 1.09 | 10.26 | 34.31 |
| %OC | **0.74** | -0.62 | 2.91 | 0.98 | 10.12 | 44.43 |
| Particle size diversity | -0.69 | **0.57** | 2.78 | 1.12 | 9.67 | 54.1 |
| Mean particle size | -0.52 | **0.43** | 2.32 | 0.49 | 8.08 | 62.18 |
| Fishing intensity | **0.29** | -0.24 | 2.31 | 0.25 | 8.05 | 70.24 |
