# Supplementary material for: Differences in meiofauna communities with sediment depth are greater than habitat effects on the New Zealand continental margin: implications for vulnerability to anthropogenic disturbance
Source: PeerJ. 2016 Jul 5;4:e2154. doi: 10.7717/peerj.2154 (PMC4941793; doi:10.7717/peerj.2154)
Supplement: Supplemental Information 3 [file peerj-04-2154-s003.docx]

Table S3. Results of PERMANOVA analysis test for the effects of habitat, water depths, sediment depth and their interaction on meiofaunal abundance at the Hikurangi Margin and Bay of Plenty study region. Significant factors at the 5% level are shown in bold. [df = degrees of freedom, SS = sum of squares, MS = mean square, Pseudo-F = Pseudo-F statistic, P = Probability, Unique perms = number of unique permutations, √ECV = square root of estimates of components of variation].

| Source | df | SS | MS | Pseudo-F | P(perm) | Unique perms | √ECV |
| --- | --- | --- | --- | --- | --- | --- | --- |
| ***Hikurangi Margin*** |  |  |  |  |  |  |  |
| **Habitat** | 2 | 395440 | 197720 | 11.761 | **0.0009** | 9959 | 86.8 |
| **Water depth** | 3 | 256260 | 85421 | 5.621 | **0.0060** | 9965 | 57.4 |
| **Sediment depth** | 1 | 485630 | 485630 | 16.608 | **0.0013** | 9844 | 89.0 |
| Habitat x Water depth | 5 | 183590 | 36717 | 2.4977 | 0.0766 | 9954 | 49.2 |
| **Habitat x Sediment depth** | 2 | 267030 | 133510 | 5.3691 | **0.0151** | 9953 | 78.9 |
| **Water depth x Sediment depth** | 3 | 1068600 | 356200 | 13.63 | **0.0004** | 9944 | 154.9 |
| **Habitat x Water depth x Sediment depth** | 5 | 427750 | 85550 | 3.5745 | **0.0225** | 9949 | 113.2 |
| Residuals | 56 | 2004600 | 35797 |  |  |  |  |
| Total | 117 | 6076700 |  |  |  |  |  |
|  |  |  |  |  |  |  |  |
| ***Bay of Plenty*** |  |  |  |  |  |  |  |
| **Habitat** | 1 | 117540 | 117540 | 10.275 | **0.0059** | 9858 | 46.4 |
| **Water depth** | 3 | 136240 | 45413 | 3.8586 | **0.0283** | 9963 | 33.2 |
| **Sediment depth** | 1 | 103300 | 103300 | 9.9835 | **0.0046** | 9831 | 36.6 |
| **Habitat x Water depth** | 3 | 129350 | 43118 | 3.3632 | **0.0383** | 9956 | 42.5 |
| Habitat x Sediment depth | 1 | 29028 | 29028 | 2.9675 | 0.1080 | 9818 | 24.2 |
| Water depth x Sediment depth | 3 | 970.06 | 323.35 | 0.24741 | 0.8632 | 9957 | -21.2 |
| Habitat x Water depth x Sediment depth | 3 | 41288 | 13763 | 1.5202 | 0.2432 | 9950 | 24.9 |
| Residuals | 92 | 1133500 | 12321 |  |  |  |  |
| Total | 141 | 2087800 |  |  |  |  |  |
