# Supplementary material for: Differences in meiofauna communities with sediment depth are greater than habitat effects on the New Zealand continental margin: implications for vulnerability to anthropogenic disturbance
Source: PeerJ. 2016 Jul 5;4:e2154. doi: 10.7717/peerj.2154 (PMC4941793; doi:10.7717/peerj.2154)
Supplement: Supplemental Information 6 — [Abundance shown in total meiofauna individuals per 10 cm2; diversity as meiofaunal taxon richness]. [file peerj-04-2154-s006.docx]

Table S6. Results of second-stage analysis for stations from 700, 1000 and 1200 m water depth strata showing meiofaunal abundance and diversity between habitats and sediment depths for Hikurangi Margin. [Abundance shown in total meiofauna individuals per 10 cm^2^; diversity as meiofaunal taxon richness].

| Stations | Abundance | Diversity |  | Abundance | Diversity | Strata (m) |
| --- | --- | --- | --- | --- | --- | --- |
| **Surface sediment (0–1 cm)** | | |  | **Subsurface sediment (1–5 cm)** | | |
| ***Canyon*** |  |  |  |  |  |  |
| 31_1 | 514 | 7 |  | 1236 | 5 | 700 |
| 31_2 | 400 | 6 |  | 2231 | 6 | 700 |
| 31_3 | 236 | 6 |  | 2467 | 7 | 700 |
| 58_3 | 418 | 7 |  | 2207 | 8 | 700 |
| 58_6 | 518 | 6 |  | 2116 | 8 | 700 |
| 58_7 | 241 | 5 |  | 2101 | 8 | 700 |
| 92_2 | 712 | 6 |  | 780 | 5 | 700 |
| 92_4 | 418 | 6 |  | 1089 | 4 | 700 |
| 27_4 | 571 | 7 |  | 1653 | 9 | 1000 |
| 27_6 | 1040 | 8 |  | 905 | 7 | 1000 |
| 27_8 | 1364 | 9 |  | 1312 | 6 | 1000 |
| 53_3 | 416 | 6 |  | 249 | 4 | 1000 |
| 62_6 | 416 | 5 |  | 771 | 5 | 1200 |
| 62_7 | 656 | 7 |  | 980 | 6 | 1200 |
| 62_8 | 403 | 6 |  | 441 | 4 | 1200 |
| 97_1 | 799 | 4 |  | 748 | 6 | 1000 |
| 98_1 | 648 | 5 |  | 641 | 3 | 1000 |
| 98_2 | 341 | 5 |  | 528 | 4 | 1000 |
| 22_1 | 752 | 6 |  | 908 | 7 | 1200 |
| 22_2 | 641 | 6 |  | 667 | 4 | 1200 |
| 22_5 | 654 | 6 |  | 1025 | 7 | 1200 |
| 127_3 | 516 | 5 |  | 827 | 3 | 1200 |
| **Average** | 576 | 6.1 |  | 1176 | 5.7 |  |
|  |  |  |  |  |  |  |
| ***Seamount*** |  |  |  |  |  |  |
| 69_1 | 369 | 6 |  | 812 | 8 | 700 |
| 69_2 | 379 | 4 |  | 443 | 2 | 700 |
| 69_5 | 430 | 5 |  | 852 | 4 | 700 |
| 72_1 | 286 | 6 |  | 639 | 5 | 1000 |
| 72_4 | 601 | 5 |  | 313 | 8 | 1000 |
| 72_8 | 160 | 6 |  | 415 | 4 | 1000 |
| 130_4 | 456 | 8 |  | 381 | 4 | 1000 |
| **Average** | 383 | 5.7 |  | 551 | 5.0 |  |
|  |  |  |  |  |  |  |
| ***Seep*** |  |  |  |  |  |  |
| 84_8_R1 | 290 | 7 |  | 1276 | 6 | 1000 |
| 84_8_R2 | 388 | 8 |  | 273 | 4 | 1000 |
| 86_9_R1 | 317 | 8 |  | 528 | 5 | 1000 |
| 86_9_R2 | 782 | 8 |  | 309 | 5 | 1000 |
| 112_10_R1 | 392 | 8 |  | 592 | 6 | 1000 |
| 112_10_R2 | 1136 | 9 |  | 669 | 7 | 1000 |
| 116_15_R1 | 1261 | 10 |  | 680 | 8 | 1000 |
| 116_15_R2 | 535 | 8 |  | 465 | 9 | 1000 |
| 118_16_R1 | 972 | 9 |  | 528 | 6 | 1000 |
| 118_16_R2 | 814 | 9 |  | 797 | 7 | 1000 |
| 123_17_R1 | 1195 | 9 |  | 546 | 7 | 1000 |
| 123_17_R2 | 840 | 6 |  | 514 | 4 | 1000 |
| **Average** | 744 | 8.3 |  | 598 | 6.2 |  |
|  |  |  |  |  |  |  |
| ***Slope*** |  |  |  |  |  |  |
| 44_4 | 580 | 6 |  | 933 | 4 | 700 |
| 44_5 | 269 | 5 |  | 814 | 7 | 700 |
| 44_7 | 575 | 6 |  | 989 | 6 | 700 |
| 124_4 | 699 | 5 |  | 773 | 5 | 700 |
| 124_7 | 861 | 6 |  | 901 | 5 | 700 |
| 4_4 | 710 | 7 |  | 997 | 5 | 1000 |
| 4_5 | 714 | 8 |  | 1095 | 6 | 1000 |
| 4_7 | 938 | 7 |  | 999 | 6 | 1000 |
| 41_1 | 328 | 5 |  | 354 | 2 | 1000 |
| 41_3 | 145 | 5 |  | 933 | 5 | 1000 |
| 41_8 | 675 | 7 |  | 548 | 7 | 1000 |
| 76_4 | 1214 | 7 |  | 671 | 6 | 1200 |
| 76_5 | 816 | 9 |  | 379 | 7 | 1200 |
| 38_2 | 1125 | 10 |  | 959 | 5 | 1200 |
| 38_3 | 599 | 6 |  | 961 | 7 | 1200 |
| 38_4 | 492 | 4 |  | 158 | 5 | 1200 |
| **Average** | 671 | 6.4 |  | 779 | 5.5 |  |
