# Supplementary material for: Differences in meiofauna communities with sediment depth are greater than habitat effects on the New Zealand continental margin: implications for vulnerability to anthropogenic disturbance
Source: PeerJ. 2016 Jul 5;4:e2154. doi: 10.7717/peerj.2154 (PMC4941793; doi:10.7717/peerj.2154)
Supplement: Supplemental Information 7 — Significant factors at the 5% level are shown in bold. [df = degrees of freedom, SS = sum of squares, MS = mean square, Pseudo-F = Pseudo-F statistic, P = Probability, Unique perms = number of unique permutations, √ECV = square root of estimates of components of variation]. [file peerj-04-2154-s007.docx]

Table S7. Results of PERMANOVA analysis tests on second stage analysis. Results showed the effects of habitat (slope, canyon, seamount and seep), water depths (700 m, 1000 m, 1200 m), sediment depth and their interaction on meiofaunal community structure at the Hikurangi Margin region, after accounting for the effect of spatial covariates (result not shown). Significant factors at the 5% level are shown in bold. [df = degrees of freedom, SS = sum of squares, MS = mean square, Pseudo-F = Pseudo-F statistic, P = Probability, Unique perms = number of unique permutations, √ECV = square root of estimates of components of variation].

| Source | df | SS | MS | Pseudo-F | P(perm) | Unique perms | √ECV |
| --- | --- | --- | --- | --- | --- | --- | --- |
| **Habitat** | 3 | 4031.7 | 1343.9 | 5.1547 | **0.0001** | 9919 | 6.6 |
| **Sediment depth** | 1 | 4532.6 | 4532.6 | 17.385 | **0.0001** | 9955 | 8.7 |
| Habitat x Sediment depth | 3 | 1170.5 | 390.16 | 1.4965 | 0.1354 | 9916 | 3.1 |
| Residuals | 104 | 27114 | 260.71 |  |  |  |  |
| Total | 113 | 38625 |  |  |  |  |  |
